# The chain mediating role of physical exercise and anxiety in the relationship between school connectedness and sleep disorders among adolescents

**DOI:** 10.3389/fpubh.2026.1881188

**Published:** 2026-06-29

**Authors:** Xiaozhen Wu, Ning Wang, Juyan Fang, Yang Liu, Binbin Zhao, Chunhui Zhou

**Affiliations:** 1 Fuyang Normal University, Fuyang, China; 2 Chengdu Vocational University of the Arts, Chengdu, China; 3 Jishou University, Jishou, China; 4 Chengdu Experimental Middle School, Chengdu, China; 5 Shenyang Normal University, Shenyang, China; 6 Zhangjiajie Institute of Aeronautical Engineering, Zhangjiajie, China

**Keywords:** adolescents, anxiety, physical exercise, school connectedness, sleep disorders

## Abstract

**Objective:**

The present study aims to investigate the chain mediating effects of Physical Exercise (PE) and anxiety in the association between School Connectedness (SC) and Sleep Disorders (SD) among adolescents.

**Methods:**

The present study employed a convenience sampling strategy to recruit 519 adolescents (Mage = 13.32 years, SD = 1.02; age range: 11–15 years) from northwestern Hunan Province, China. Self-report measures were administered to assess SC, SD, PE, and anxiety. Descriptive statistics, bivariate correlation analyses, and mediation effect examinations were conducted using SPSS (version 26.0) in conjunction with the PROCESS macro (Model 6).

**Results:**

After adjusting for age and sex, the findings revealed that SC was significantly and inversely correlated with both PE and SD. Moreover, SC demonstrated a significant negative association with anxiety and SD. Additionally, PE exhibited a significant inverse relationship with anxiety. SC was correlated with SD among adolescents, and both anxiety and PE were also correlated with SD.

**Conclusion:**

The current study provided additional insight into the psychological pathways linking SC and SD in adolescents, specifically by exploring how PE and anxiety serve as mediating factors in this association.

## 1 Background

Adolescence is characterized by substantial physiological and psychological transformations, during which Sleep Disorderss (SD) have emerged as an increasingly prevalent public health concern (1). SD encompass abnormalities in sleep quality, duration, or circadian patterns, with clinical manifestations including insomnia, sleep-disordered breathing, and circadian rhythm disturbances (2). These disorders are particularly common among adolescents, often presenting as hyperactivity, inattention, irritability, or oppositional behaviors rather than classic somnolence (3). Epidemiological evidence indicates that the prevalence of SD among youth remains elevated worldwide. A survey of 3,443 middle school students in eastern coastal China reported an overall prevalence of 10.8%, with significantly higher rates observed in females relative to males (4). Additionally, a prior investigation documented a prevalence of approximately 26% among Chinese adolescents (5); such discrepancies across studies may be attributable to variations in assessment instruments and sample characteristics. From a physiological perspective, adolescence is characterized by significant neuroendocrine remodeling and reorganization of the circadian rhythm system. Research has shown that the pubertal stage is associated with a physiological delay in the phase of endogenous melatonin secretion, which, combined with heightened sensitivity to nocturnal light exposure, leads to a postponed sleep onset time (6). Carskadon's “perfect storm” model proposes that the interaction among biological regulatory pressure, psychosocial stress, and school environmental demands collectively forms the basis for the elevated prevalence of sleep problems during adolescence (7). Furthermore, from a neurodevelopmental standpoint, the adolescent brain undergoes marked synaptic pruning and myelination processes. The prefrontal cortex matures later than the limbic system, and this developmental asynchrony results in relatively insufficient emotional regulation capacity. Sleep plays a critical supportive role in this neural remodeling process (8). From a psychological perspective, sleep disturbances may lead to negative emotional or behavioral problems in adolescents, contributing to developmental delays and accidental injuries (9). A systematic review further revealed that SD in youth are associated with multiple adverse outcomes, including psychological distress, anxiety, depression, and excessive reliance on electronic devices (10). In addition, alterations in sleep architecture, together with external factors—particularly the influence of circadian rhythms and social demands—may drive adolescents toward insufficient sleep or sleep deficiency (11). Collectively, given the detrimental impact of SD on adolescent PE and mental wellbeing, elucidating the underlying mechanisms is imperative for informing the development of targeted intervention strategies.

### 1.1 School connectedness, sleep disorders

Schools serve as the primary contexts for adolescent learning, daily living, and social interaction, with their environmental characteristics exerting profound influences on youth physical and psychological development (12). SC refers to the degree to which students perceive themselves as accepted, included, respected, and supported by teachers and peers within the school environment. It reflects students‘ positive attitudes toward school, their sense of belonging, and their affective engagement with others in the school context (13). SC constitutes a critical indicator of adolescent social adaptation and psychological wellbeing, with higher levels associated with reduced internalizing conditions such as anxiety and depression, thereby fostering positive developmental trajectories (14). Recently, as the detection rate of SD among adolescents has continued to escalate, the relationship between SC and sleep health has garnered increasing scholarly attention (15). Based on the cognitive-behavioral model (CBM) (16), the school environment and social schedules constitute core determinants of adolescent circadian regularity. Students who report a high level of SC are more inclined to adhere to institutional routines, thereby maintaining consistent sleep–wake patterns. In contrast, those with low SC may engage in “revenge bedtime procrastination”—a behavior aimed at compensating for insufficient daytime social interaction and autonomy—to regain a sense of psychological control. This behavioral pattern often results in difficulty initiating sleep, nocturnal awakenings, and excessive daytime sleepiness, ultimately culminating in clinically significant sleep disturbances. A systematic review of longitudinal investigations demonstrated that adolescents' school performance, school-related psychological factors, and structural elements exert sustained effects on youth health outcomes, most likely including their sleep patterns (i.e., sleep quality and SD) (17). From a protective factors perspective (18), SC functions as a significant safeguard for sleep quality; by enhancing feelings of belonging, social support, and peer support, it may improve adolescent sleep quality to a certain extent and reduce the incidence of SD. In a longitudinal study of 502 adolescents entering high school, Brodar et al. (19) identified a significant association between peer pressure and insomnia symptoms, with this relationship being particularly pronounced among female adolescents. Specifically, peer pressure indirectly influenced both concurrent and subsequent insomnia symptoms in females through the mediating role of repetitive negative thinking. Concurrently, a systematic review examined the associations between social anxiety and multiple sleep parameters in adolescents, including sleep quality, sleep disturbances, and sleep duration (20). On this basis, the present study proposes Hypothesis 1: School Connectedness is significantly associated with Sleep Disorders among adolescents.

### 1.2 School connectedness, physical exercise, sleep disorders

Conventionally, PE refers to skeletal-muscle-driven bodily movement that consumes energy, with aerobic exercise representing one of its forms, resistance training, and recreational sports, among other modalities (21). Schools that actively organize various forms of PE provide students not only with a platform to demonstrate their talents and cultivate team spirit (22) but also an opportunity to enhance Classmate Support (CS) and establish close Teacher Support (TS) through these activities (23). A longitudinal study conducted between 2016 and 2020 found that changes in perceived SC were significantly associated with changes in PE levels. Moreover, SC played a significant protective role in mitigating the decline in PE levels associated with increasing grade levels, particularly among female adolescents (24). A study from Latvia further revealed a positive correlation between a supportive school climate and high levels of participation in PE (25). Self-Determination Theory asserts that meeting the fundamental psychological needs for autonomy, competence, and relatedness facilitates intrinsic motivation and sustained behavioral engagement (26). When schools actively facilitate PE, they fulfill students' relatedness needs (team belonging) and competence needs (skill acquisition), thereby strengthening their intrinsic motivation to participate in PE (27). A substantial body of research indicates that engaging in consistent PE facilitates muscle relaxation, alleviates negative emotions, and regulates circadian rhythms and core body temperature, thereby exerting a positive impact on sleep outcomes (28, 29). Furthermore, physical activity may improve sleep quality by modulating sleep-related neurotransmitters, such as optimizing dopamine levels (30). Based on this foundation, the current study puts forward Hypothesis 2: Physical Exercise mediates the relationship between School Connectedness and Sleep Disorders among adolescents.

### 1.3 School connectedness, anxiety, sleep disorders

The period of adolescence is a crucial transitional phase during which substantial physical and psychological maturation occurs, during which mental health status is susceptible to the interplay of internal and external factors. According to the systematic development theory (31), positive human development arises from bidirectional and mutually beneficial interactions between individuals and their environmental resources. Within this framework, schools serve as a core developmental context for adolescents, and the connective resources provided by schools directly influence individuals' psychological adaptation. Anxiety is a highly prevalent mental disorder resulting from a complex interplay of social, psychological, and biological factors (32). The biopsychosocial model further contends that interactions across biological, psychological, and social domains give rise to negative emotional outcomes, with adolescents' emotional experiences in school shaped by cognitive appraisals and social support (33). Most scholars agree that low SC serves as a risk factor for anxiety. This association may be explained by the tendency of students with low levels of connectedness to perceive negative social evaluations—such as being excluded or ridiculed—which can trigger social anxiety (34). Furthermore, a lack of care and acceptance from teachers and peers, coupled with an absence of perceived safety and fairness, deprives students of buffering resources when facing stress, thereby contributing to the development of anxiety (35–37). Empirical evidence indicates that a strong sense of school belonging (SB) and positive peer relationships significantly reduce adolescent anxiety (38). The close association between anxiety and SD has been extensively documented. Epidemiological data reveal that most adolescents with anxiety present with at least one sleep-related complaint, comprising difficulties in initiating sleep, sustaining sleep, or experiencing early morning awakenings (39). Anxiety may impair sleep through multiple mechanisms: cognitive rumination adversely affects sleep quality; physiological hyperarousal disrupts sleep continuity (40, 41); and behavioral irregularities, such as disrupted daily routines, shorten sleep duration (42). These findings suggest that anxiety may influence adolescent SD through SC. Accordingly, we posit Hypothesis H3: anxiety serves as a mediator between School Connectedness and adolescent sleep disorders.

### 1.4 Physical exercise, anxiety

PE is widely recognized as a core component of a healthy lifestyle. Extensive evidence supports that regular PE both protects physical health and significantly enhances mental wellbeing (43). According to biopsychological theory (44), Through activation of the hypothalamic–pituitary–adrenal (HPA) axis and the sympathetic–parasympathetic nervous system, PE enhances both the secretion and metabolism of key neurotransmitters, namely dopamine, endorphins, and serotonin. These agents are critically involved in affect regulation, stress relief, and reward processing. Through consistent engagement in PE, adolescents can elevate the baseline levels of these neurotransmitters and enhance prefrontal cortex regulation over the limbic system, thereby improving emotional states and reducing anxiety (45). As demonstrated by additional studies, adolescents who engage in longer sessions of moderate-intensity PE exhibit significantly lower anxiety levels; in other words, greater exercise duration at moderate intensity is linked to diminished anxiety severity (46). Additionally, studies have established a strong link between PE and adolescent mental health (47, 48), showing that PE significantly reduces anxiety symptoms (49). The stress-buffering model posits that social assets, including SC, can attenuate the negative impact of stressful experiences, indirectly protect physical and mental health through two pathways: promoting adaptive behaviors (e.g., PE) and mitigating maladaptive psychological states (e.g., anxiety) (50). Based on this framework, the present study proposes Hypothesis H4: Physical Exercise and anxiety serve as serial mediators in the relationship between School Connectedness and adolescent Sleep Disorders.

#### 1.5 Overview

**Figure 1:**
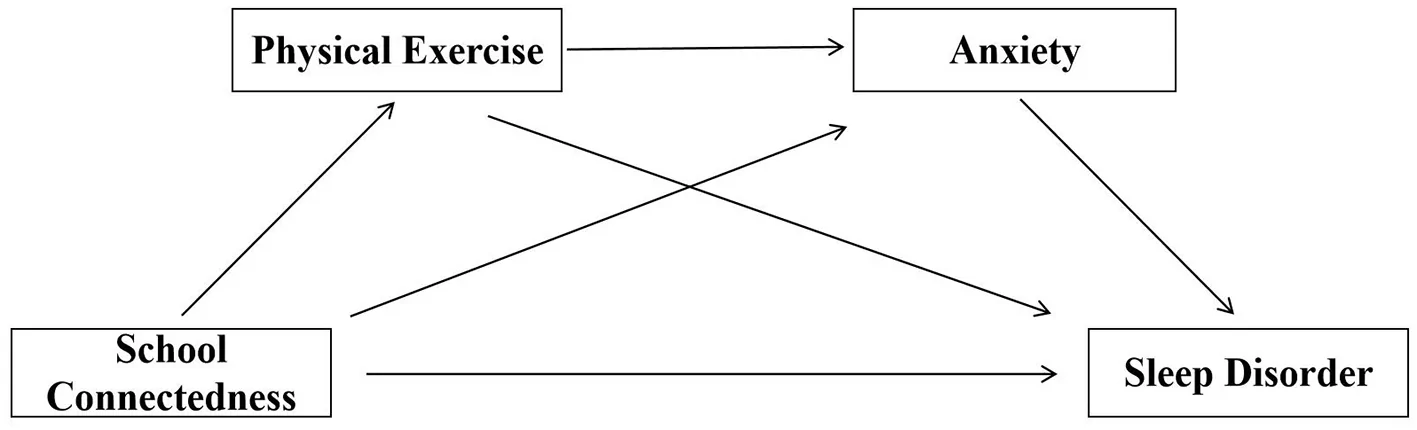
Chain mediation hypothesis model.

Drawing on the theoretical and empirical grounds discussed above, the current study uses PE and anxiety as sequential mediators to further clarify the psychological and behavioral pathways through which SC relates to SD in adolescents. Consequently, a serial mediation model was established (as depicted in Figure 1), incorporating PE and anxiety as mediating variables to investigate the relationship between SC and adolescent SD.

## 2 Methods

### 2.1 Participates

This study was conducted in December 2025, employing a sample of 616 adolescents drawn from three secondary schools in Hunan Province. Through convenience sampling procedures, participants filled out questionnaires measuring SC, SD, PE, and anxiety levels. Before data collection began, the research team clearly outlined the study's objectives and reaffirmed their guarantee of participant anonymity, data confidentiality, and the potential positive implications of the research. Furthermore, all participants were explicitly informed of their absolute right to discontinue participation at any point without facing any adverse consequences, thereby safeguarding their ethical autonomy and rights. Ethical approval for the research was obtained from the institutional biomedical ethics committee before the study began, and written informed consent was collected from both participants and their guardians to ensure adherence to ethical standards. After data collection, a rigorous screening process eliminated incomplete or patterned responses, yielding 519 valid questionnaires (277 boys, 242 girls). The ages of respondents ranged from 11 to 15 years, with a mean age of 13.32 (SD = 1.02).

### 2.2 School connectedness

This research employed the “School Connectedness Scale,” revised by Yu Chengfu et al. (51). This measure employed a 5-point Likert scale, with responses ranging from 1 (not at all) to 5 (very much), such that higher scores denote greater levels of perceived SC. In this study, the Cronbach's α coefficient for the overall scale was 0.803. For the three dimensions—TS, CS, and SB—the Cronbach's α coefficients were 0.631, 0.638, and 0.733, respectively. It should be noted that prior research has established that when a variable is measured with fewer than six observations, a Cronbach's α coefficient greater than 0.6 indicates acceptable reliability of the scale (52).

### 2.3 Physical exercise

The research employed the “Physical Exercise Level Scale,” revised by Liang Deqing and his team (53). This scale measures PE across three core dimensions—intensity, duration, and frequency—using the following scoring formula: Intensity × (Duration - 1) × Frequency = Total Physical Exercise Score. A higher composite score reflects greater PE engagement. In this sample, the Cronbach's α coefficient was computed as 0.715.

### 2.4 Anxiety

This study employed the anxiety subscale of the Depression-Anxiety-Stress Scale (DASS-21) as revised by Gong Xu and his colleagues (54). This subscale consists of seven items, each rated on a 4-point scale ranging from 1 (not at all true) to 4 (very much true), whereby higher scores indicate greater anxiety severity. The subscale achieved a Cronbach's alpha of 0.761 for the current sample.

### 2.5 Sleep disorders

The sleep disturbance component of the Pittsburgh Sleep Quality Index, originally created by Buysse et al. at the University of Pittsburgh, was utilized in the current research (55). This subscale consists of nine items, with responses scored from 1 (none) to 4 (three times or more per week). The scoring protocol awards 0 points for “none,” 1 point for “less than once per week,” 2 points for “one to two times per week,” and 3 points for “more than three times per week.” Elevated scores reflect a greater occurrence of sleep disturbances, with total scores ranging from 0 to 27. For the present sample, the subscale yielded a Cronbach's alpha of 0.760.

### 2.6 Statistical analysis

Statistical analyses were conducted using SPSS version 26.0. Initially, potential common method bias was evaluated, with results indicating no significant bias as the observed value fell below the 40% threshold (56). Descriptive statistics and correlation analyses were then conducted to examine the demographic characteristics and primary variables of the sample. Prior to further modeling, all key variables were standardized. Hypothesis testing was carried out using the PROCESS macro (Model 6) in SPSS to investigate the association between SC and adolescent SD, with particular emphasis on the chain-mediating roles of PE and anxiety (57). The model's fit was evaluated, and 95% confidence intervals (95% CI) were calculated using 5,000 bootstrap iterations to ensure the robustness of the analysis (58). Gender and age were entered as covariates, and statistical significance was established at α = 0.05.

## 3 Results

### 3.1 Common method deviation test

In the present study, common method bias testing identified two factors with eigenvalues exceeding 1. The first factor explained 37.19% of the total variance, falling below the 40% threshold. These findings suggest that common method bias does not present a significant threat to the validity of the results.

### 3.2 Description analysis

As shown in Table 1, significant gender differences were observed in SC (*t* = 3.47, *p* < 0.01), PE (*t* = 7.46, *p* < 0.001), anxiety (*t* = −4.06, *p* < 0.001), and SD (*t* = −3.36, *p* < 0.01). Specifically, males reported higher levels of SC and PE than females, whereas females exhibited higher levels of anxiety and SD compared to males. Regarding the three dimensions of SC, significant gender differences were found in TS (*t* = 4.04, *p* < 0.001) and SB (*t* = 3.08, *p* < 0.01), with males scoring higher than females in both dimensions.

**Table 1:** Describes the analysis.

Variables	School connectedness		Teacher support		Classmate support		School belonging		Anxiety		Physical exercise		Sleep disorder	
	Mean	Sd	Mean	Sd	Mean	Sd	Mean	Sd	Mean	Sd	Mean	Sd	Mean	Sd
Boys	27.02	5.76	8.05	1.90	10.34	2.42	8.63	2.55	11.88	4.32	25.88	23.55	5.32	4.84
Girls	25.36	5.07	7.41	1.63	9.97	2.19	7.97	2.31	13.42	4.27	13.32	14.19	6.73	4.67
*t*	3.47^**^	4.04^***^	1.81	3.08^**^	−4.06^***^	7.46^***^	−3.36^**^

^**^p < 0.01; ^***^p < 0.001.

### 3.3 Correlation analysis

The results presented in Table 2 indicate that SC is positively correlated with PE, and negatively correlated with both anxiety and SD. PE is negatively correlated with anxiety and SD, whereas anxiety is positively correlated with SD. Regarding the three dimensions of SC, the correlations are as follows: TS is positively correlated with SC, CS, SB, and PE, and negatively correlated with SD. CS is positively correlated with SC and SB, and negatively correlated with SD. SB is positively correlated with SC and PE, and negatively correlated with both anxiety and SD.

**Table 2:** Correlation analysis.

Variables	1	2	3	4	5	6	7	8
1 Age	–							
2 School connectedness	−0.054	–						
3 Teacher support	−0.089^*^	0.796^***^	–					
4 Classmate support	−0.052	0.834^***^	0.505^***^	–				
5 School belonging	−0.005	0.867^***^	0.571^***^	0.554^***^	–			
6 Physical exercise	0.097^*^	0.139^**^	0.100^*^	0.044	0.197^***^	–		
7 Anxiety	−0.007	−0.158^***^	−0.058	−0.035	−0.277^***^	−0.168^***^	–	
8 Sleep disorder	−0.075	−0.259^***^	−0.141^**^	−0.150^**^	−0.335^***^	−0.194^***^	0.583^***^	–

^*^ *p* < 0.05; ^**^ *p* < 0.01:^***^: *p* < 0.001.

### 3.4 Mediation model testing

**Figure 2:**
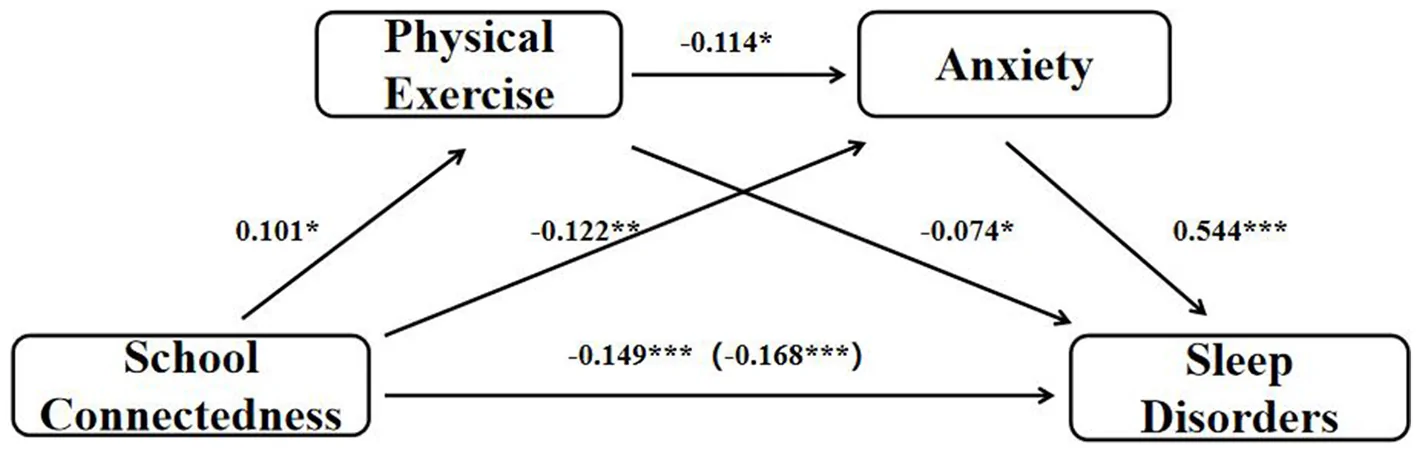
Diagram of the chain mediation model. *p < 0.05; **p < 0.01; ***p < 0.001.

After controlling for demographic variables, the results presented in Table 3 indicate that SC was negatively associated with SD (β = −0.249, *p* < 0.001). After the inclusion of mediating variables, the association between SC and SD remained significant (β = −0.168, *p* < 0.001). Furthermore, examination of the mediation model revealed a positive association between SC and PE (β = 0.101, *p* < 0.05), whereas PE was negatively associated with SD (β = −0.074, *p* < 0.05). SC was negatively associated with anxiety (β = −0.122, *p* < 0.01), and anxiety was positively associated with SD (β = 0.544, *p* < 0.001). Finally, PE and anxiety served as serial mediators in the relationship between SC and adolescent SD (β = −0.114, *p* < 0.05). The proportions of the mediating effects are presented in Table 4, and the serial mediation model is illustrated in Figure 2.

**Table 3:** Tests the mediation model.

Outcome variable	Predictive variable	β	SE	*t*	*R* ^2^	*F*
Sleep disorder	Gender	0.100	0.043	2.331^*^	0.085	15.945^***^
	Age	−0.078	0.043	−1.828		
	School connectedness	−0.249	0.043	−5.815^***^		
Physical exercise	Gender	−0.280	0.042	−6.590^***^	0.106	20.299^***^
	Age	0.072	0.042	1.706		
	School connectedness	0.101	0.042	2.38^*^		
Anxiety	Gender	0.124	0.045	2.736^**^	0.060	8.225^***^
	Age	0.011	0.043	0.254		
	School connectedness	−0.122	0.044	−2.809^**^		
	Physical exercise	−0.114	0.045	−2.520^*^		
Sleep disorder	Gender	−0.006	0.037	−0.149	0.380	62.919^***^
	Age	−0.074	0.035	−2.103^*^		
	School connectedness	−0.168	0.036	−4.707^***^		
	Physical eExercise	−0.074	0.037	−2.003^*^		
	Anxiety	0.544	0.036	15.184^***^		

^*^ *p* < 0.05; ^**^ *p* < 0.01; ^***^: *p* < 0.001.

**Table 4:** Path analysis of mediation model.

Intermediate path	Effect size	SE	Bootstrap 95% CI	Proportion of mediating effect
Gross effect	−0.249	0.036	−0.333,−0.165	
Direct effect	−0.168	0.036	−0.238,−0.098	
School connectedness → Physical exercise → Sleep disorder	−0.007	0.006	−0.021,−0.001	2.811%
School connectedness → Anxiety → Sleep disorder	−0.067	0.033	−0.135,−0.005	26.907%
School connectedness → Physical exercise → Anxiety → Sleep disorder	−0.006	0.004	−0.015,−0.001	2.410%
Total indirect effect	−0.080	0.034	−0.151,−0.018	32.129%

**Figure 3:**
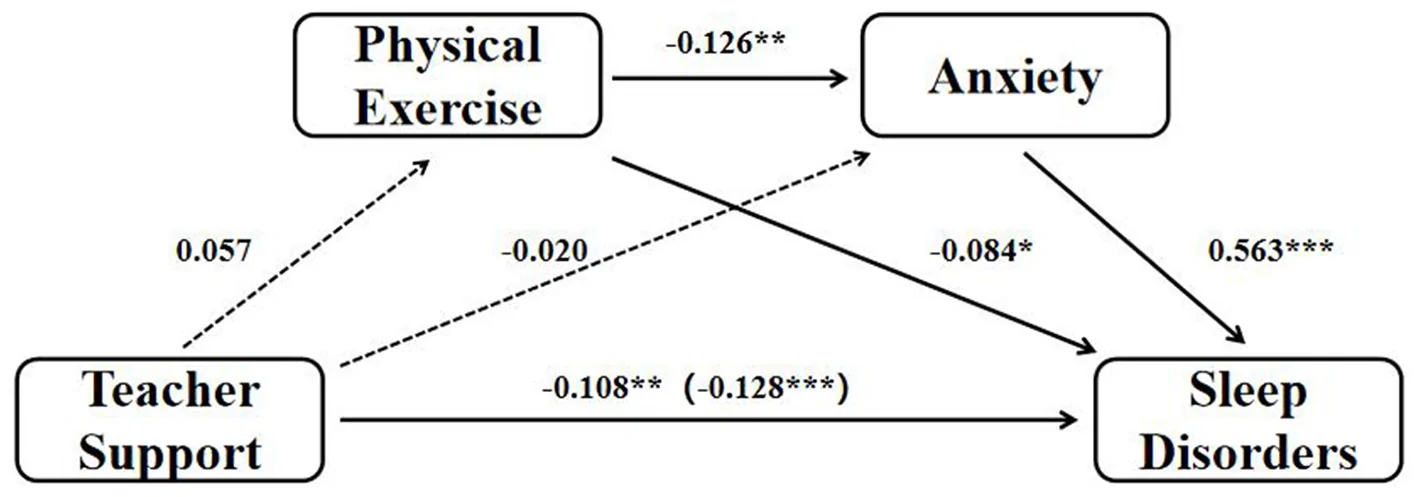
Diagram of the chain mediation model. *p < 0.05; **p < 0.01; ***p < 0.001.

After controlling for demographic variables, the TS dimension was examined separately. As shown in Table 5, TS was negatively associated with SD (β = −0.128, *p* < 0.01). After the inclusion of the two mediating variables, the association between TS and SD remained significant (β = −0.108, *p* < 0.01). Furthermore, mediation analysis revealed that TS was not significantly associated with PE, whereas PE was negatively associated with SD (β = −0.084, *p* < 0.05). Similarly, TS showed no significant association with anxiety, while anxiety was positively associated with SD (β = 0.563, *p* < 0.001). Finally, PE and anxiety acted as serial mediators in the relationship between TS and adolescent SD (β = −0.126, *p* < 0.01). The proportions of the mediating effects are presented in Table 6, and the serial mediation model is illustrated in Figure 3.

**Table 5:** Tests the mediation model.

Outcome variable	Predictive variable	β	SE	*t*	*R* ^2^	*F*
Sleep disorder	Gender	0.116	0.044	2.615^**^	0.041	7.258^***^
	Age	−0.074	0.044	−1.696		
	Teacher support	−0.128	0.044	−2.898^**^		
Physical exercise	Gender	−0.285	0.043	−6.658^***^	0.099	18.839^***^
	Age	0.071	0.042	1.670		
	Teacher support	0.057	0.043	1.322		
Anxiety	Gender	0.136	0.046	2.962^**^	0.046	6.213^***^
	Age	0.018	0.044	0.418		
	Teacher support	−0.020	0.044	−0.458		
	Physical exercise	−0.126	0.045	−2.778^**^		
Sleep disorder	Gender	−0.005	0.038	−0.136	0.364	58.831^***^
	Age	−0.074	0.035	−2.103^*^		
	Teacher support	−0.108	0.036	−2.990^**^		
	Physical exercise	−0.084	0.037	−2.236^*^		
	Anxiety	0.563	0.036	15.622^***^		

^*^ *p* < 0.05; ^**^ *p* < 0.01; ^***^ *p* < 0.001.

**Table 6:** Path analysis of mediation model.

Intermediate path	Effect size	SE	Bootstrap 95% CI	Proportion of mediating effect
Gross effect	−0.128	0.044	−0.215,−0.041	
Direct effect	−0.108	0.036	−0.179,−0.037	
Teacher support → Physical exercise → Sleep disorder	−0.005	0.005	−0.016,−0.003	3.906%
Teacher support → Anxiety → Sleep disorder	−0.011	0.028	−0.069,−0.041	8.593%
Teacher support → Physical exercise → Anxiety → Sleep disorder	−0.004	0.004	−0.012,−0.002	3.125%
Total indirect effect	−0.020	0.030	−0.081,−0.036	15.625%

**Figure 4:**
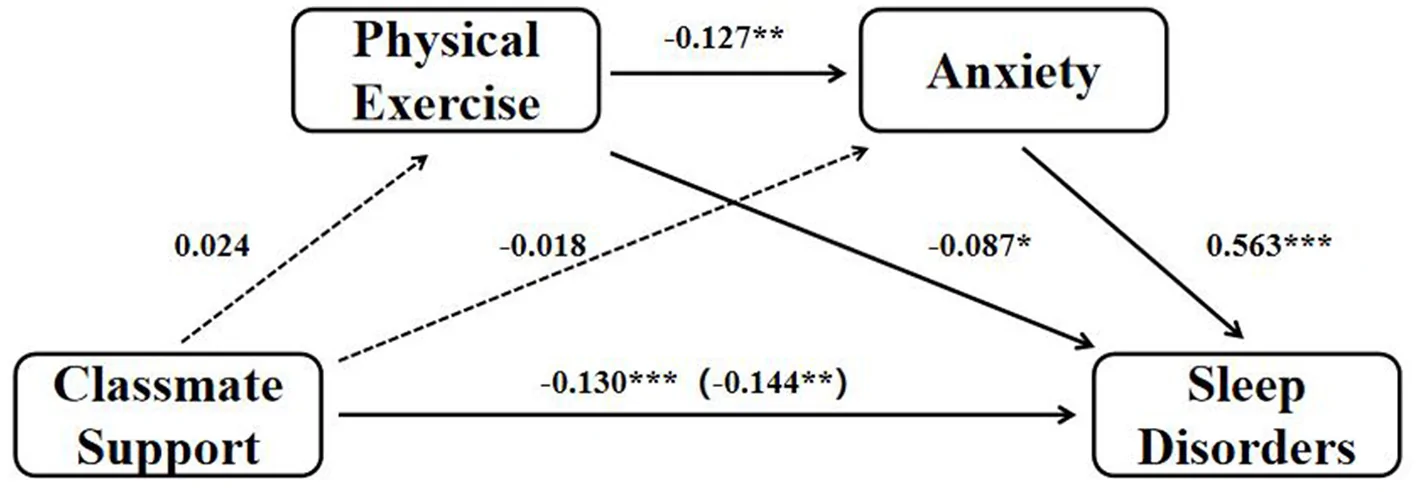
Diagram of the chain mediation model. *p < 0.05; **p < 0.01; ***p < 0.001.

After controlling for demographic variables, the CS dimension was examined separately. As shown in Table 7, CS was negatively associated with SD (β = −0.144, *p* < 0.01). After the inclusion of the two mediating variables, the association between CS and SD remained significant (β = −0.130, *p* < 0.001). Furthermore, mediation analysis revealed that CS was not significantly associated with PE, whereas PE was negatively associated with SD (β = −0.087, *p* < 0.05). Similarly, CS showed no significant association with anxiety, while anxiety was positively associated with SD (β = 0.563, *p* < 0.001). Finally, PE and anxiety acted as serial mediators in the relationship between CS and adolescent SD (β = −0.127, *p* < 0.01). The proportions of the mediating effects are presented in Table 8, and the serial mediation model is illustrated in Figure 4.

**Table 7:** Tests the mediation model.

Outcome variable	Predictive variable	β	SE	*t*	*R* ^2^	*F*
Sleep disorder	Gender	0.127	0.043	2.925^**^	0.045	8.147^***^
	Age	−0.069	0.043	−1.588		
	Classmate support	−0.144	0.043	−3.317^**^		
Physical exercise	Gender	−0.294	0.042	−6.944^***^	0.096	18.315^***^
	Age	0.066	0.042	1.563		
	Classmate support	0.024	0.042	0.571		
Anxiety	Gender	0.138	0.045	3.038^**^	0.046	6.202^***^
	Age	0.019	0.044	0.447		
	Classmate support	−0.018	0.043	−0.410		
	Physical exercise	−0.127	0.045	−0.410^**^		
Sleep disorder	Gender	−0.003	0.037	−0.080	0.370	60.259^***^
	Age	−0.069	0.035	−1.960^*^		
	Classmate support	−0.130	0.035	−3.682^***^		
	Physical exercise	−0.087	0.037	−2.331^*^		
	Anxiety	0.563	0.036	15.622^***^		

^*^ *p* < 0.05; ^**^ *p* < 0.01; ^***^ *p* < 0.001.

**Table 8:** Path analysis of mediation model.

Intermediate path	Effect size	SE	Bootstrap 95% CI	Proportion of mediating effect
Gross effect	−0.144	0.043	−0.229,−0.059	
Direct effect	−0.130	0.035	−0.199,−0.061	
Classmate support → Physical exercise → Sleep disorder	−0.002	0.005	−0.013,−0.007	1.389%
Classmate support → Anxiety → Sleep disorder	−0.010	0.031	−0.074,−0.048	6.944%
Classmate support → Physical exercise → Anxiety → Sleep disorder	−0.002	0.004	−0.010,−0.005	1.389%
Total indirect effect	−0.014	0.031	−0.080,−0.046	9.722%

**Figure 5:**
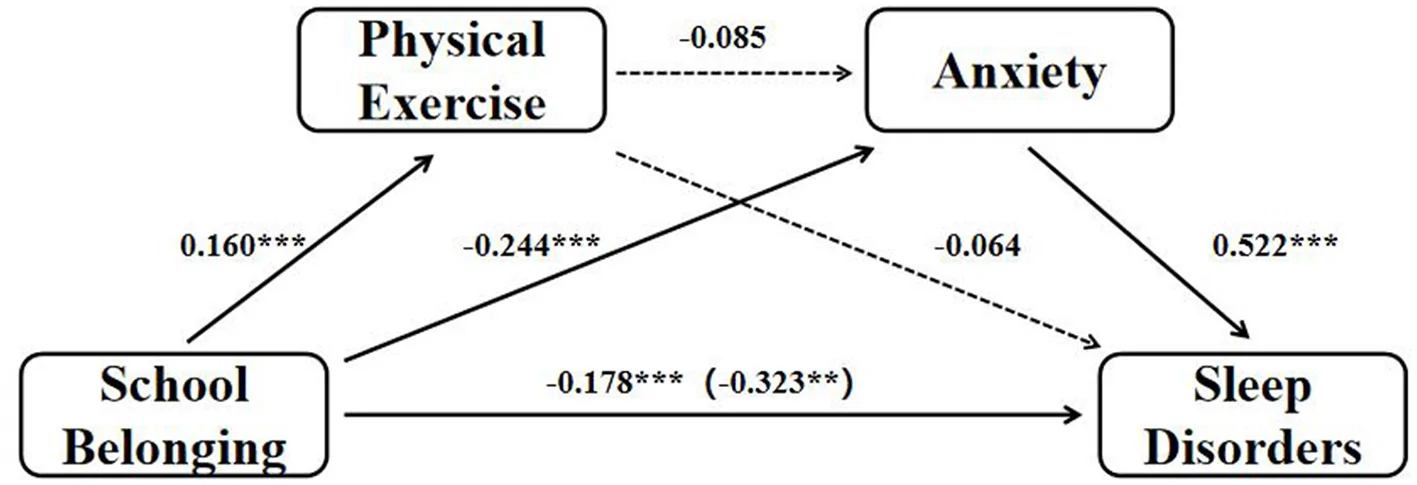
Diagram of the chain mediation model. **p < 0.01; ***p < 0.001.

After controlling for demographic variables, the SB dimension was examined separately. As shown in Table 9, SB was negatively associated with SD (β = −0.323, *p* < 0.001). After the inclusion of the two mediating variables, the association between SB and SD remained significant (β = −0.178, *p* < 0.001). Furthermore, mediation analysis revealed that SB was positively associated with PE (β = 0.160, *p* < 0.001), whereas PE showed no significant association with SD. SB was negatively associated with anxiety (β = −0.244, *p* < 0.001), while anxiety was positively associated with SD (β = 0.522, *p* < 0.001). Finally, PE and anxiety did not exhibit a significant serial mediating effect in the relationship between SB and adolescent SD. The proportions of the mediating effects are presented in Table 10, and the serial mediation model is illustrated in Figure 5.

**Table 9:** Tests the mediation model.

Outcome variable	Predictive variable	β	SE	*t*	*R* ^2^	*F*
Sleep disorder	Gender	0.096	0.042	2.285^*^	0.127	25.049^***^
	Age	−0.067	0.041	−1.607		
	School belonging	−0.323	0.042	−7.775^***^		
Physical exercise	Gender	−0.274	0.042	−6.530^***^	0.121	23.657^***^
	Age	0.068	0.042	1.630		
	School belonging	0.160	0.042	3.849^***^		
Anxiety	Gender	0.119	0.044	2.693^**^	0.103	14.690^***^
	Age	0.013	0.042	0.306		
	School belonging	−0.244	0.043	−5.707^***^		
	Physical exercise	−0.085	0.045	−1.901		
Sleep disorder	Gender	0.004	0.037	−0.103	0.382	63.386^***^
	Age	−0.066	0.035	−1.883		
	School belonging	−0.178	0.037	−4.865^***^		
	Physical exercise	−0.064	0.037	−1.727		
	Anxiety	0.522	0.037	14.237^***^		

^*^ *p* < 0.05; ^**^ *p* < 0.01; ^***^ *p* < 0.001.

**Table 10:** Path analysis of mediation model.

Intermediate path	Effect size	SE	Bootstrap 95% CI	Proportion of mediating effect
Gross effect	−0.323	0.042	−0.405,−0.241	
Direct effect	−0.178	0.037	−0.250,−0.106	
School belonging → Physical exercise → Sleep disorder	−0.010	0.007	−0.026,−0.001	3.095%
School belonging → Anxiety → Sleep disorder	−0.127	0.030	−0.191,−0.072	39.318%
School belonging → Physical exercise → Anxiety → Sleep disorder	−0.007	0.004	−0.016,−0.0001	2.167%
Total indirect effect	−0.145	0.031	−0.210,−0.088	44.891%

## 4 Discussion

The present investigation examined the interrelationships among SC, SD, PE, and anxiety. SC demonstrated a significant inverse association with SD among adolescents, and this association remained statistically significant after accounting for the mediating variables of PE and anxiety. Moreover, PE and anxiety jointly functioned as sequential mediators in the association between SC and adolescent SD. The findings offer crucial theoretical bases and practical insights that can advance the study of psychological and behavioral health among adolescents.

### 4.1 Association between school connectedness and sleep disorders

The present investigation revealed a significant inverse association between SC and SD among adolescents, a finding consistent with prior empirical evidence (59). Specifically, adolescents exhibiting elevated SC demonstrated markedly lower incidence rates of SD relative to their counterparts with diminished connectedness, underscoring the protective function of SC in safeguarding adolescent sleep health. From the standpoint of ecological systems theory (60), schools represent the central microsystem within which adolescents are situated, and the quality of connectedness within this context exerts direct influence on developmental adaptation. Students who perceive strong SBness demonstrate enhanced capacity to integrate into the educational environment and establish stable, supportive relationships with peers and educators. Such constructive social bonds serve to buffer the psychological burden stemming from academic competition and interpersonal conflict, thereby reducing maladaptive costs and mitigating sleep-onset difficulties and sleep maintenance disturbances precipitated by emotional distress. Neurobiological investigations have further elucidated that positive social relationships facilitate oxytocin secretion, modulate hypothalamic-pituitary-adrenal axis functioning, suppress excessive cortisol release, and enhance the activity of neurotransmitters including serotonin and gamma-aminobutyric acid; these biochemical alterations contribute to circadian rhythm stabilization, shortened sleep latency, and prolonged slow-wave sleep duration (61). The present findings additionally demonstrated that adolescents within high-SC groups faced significantly attenuated risk for SD, with the quality of peer support exhibiting close associations with sleep parameters. Collectively, these lines of evidence indicate that SC represents not merely a critical indicator of adolescent social adaptation, but also a modifiable protective factor for sleep health amenable to intervention.

### 4.2 School connectedness, physical exercise, sleep disorders

Consistent with prior empirical findings (62), the present investigation revealed that PE mediates the association between SC and SD among adolescents. Self-Determination Theory (26) fprovides a comprehensive framework for explaining this mediating mechanism, asserting that satisfying three basic psychological needs—autonomy, competence, and relatedness—forms the intrinsic motivational drive that underlies persistent behavioral engagement. The sense of belonging and positive affective interactions inherent in SC serve as critical socio-psychological resources that facilitate adolescents' continued participation in PE. When SC is diminished, adolescents are deprived of the social context necessary for sharing athletic experiences with peers and educators, thereby precluding the emotional rewards and identity affirmation derived from such engagement and consequently attenuating intrinsic motivation for PE (27). From the perspective of social support, peers constitute a core component of adolescents' social support networks. Engaging in PE creates shared social contexts that offer adolescents opportunities to build trust, express emotions, and gain recognition. According to the buffering model of social support proposed by Cohen and colleagues, adequate CS can mitigate the psychological impact of stressful events (63). The present findings further demonstrated a significant inverse association between PE levels and SD among adolescents. Regular PE exerts protective effects on sleep architecture and quality through multiple pathways, including enhanced energy expenditure, alleviation of accumulated diurnal stress, and modulation of biological rhythms. Relevant research indicates that participation in leisure-time PE, team sports, and activities such as table tennis and badminton can effectively alleviate psychological distress and SD, while also playing a significant role in enhancing individuals' positive psychological wellbeing (64). Therefore, strengthening emotional bonds and interactive support among teachers, peers, and students serves to elevate the initiative and sustainability of PE engagement, thereby activating the dual physiological and psychological regulatory functions of PE and ultimately reducing the risk of SD.

### 4.3 School connectedness, anxiety, sleep disorders

The current study found that anxiety serves as a mediator in the relationship between SC and SD in adolescents, which aligns with previous empirical findings (65). From the perspective of social support, schools serve as the primary setting where adolescents engage in peer interactions. Adolescents with higher levels of School Connectedness tend to perceive stronger positive bonds with the school environment, including teachers and peers. Such perceived connectedness enhances their positive beliefs and cognitive appraisals regarding key interpersonal figures—namely classmates and teachers—while reducing irrational concerns and avoidance behaviors in social situations. Consequently, this process lowers the subsequent risk of social anxiety. The quality of social interactions within the school environment is intimately linked to students' psychological wellbeing and emotional functioning. Neuroimaging investigations have demonstrated that emotional exchange with peers and the sense of belonging modulate prefrontal cortical processing of sensory information, thereby generating appropriate emotional responses (66). When SC is elevated, students are able to obtain sustained emotional support and validation from peers and educators; such positive social experiences facilitate adaptive cognitive restructuring, inhibit catastrophizing cognitions and excessive worry, and thereby attenuate anxiety. Conversely, diminished SC renders adolescents vulnerable to excessive academic pressure within a context of social deprivation, resulting in the accumulation of psychological distress that elevates anxiety levels and subsequently precipitates SD. According to the neurobiological theory of sleep, the generation of sleep and wakefulness states is closely associated with changes in neural nuclei and neuronal activity within the brain (67). A cross-sectional investigation further documented that anxiety significantly disrupts sleep initiation and maintenance processes through the activation of rumination, with anxiety exhibiting a significant positive association with SD (68). Meanwhile, individuals with anxiety may experience emotion regulation deficits due to insufficient sleep, while pre-existing emotional disturbances can also lead to disruptions in sleep architecture. This bidirectional relationship creates a vicious cycle in which anxiety and SD mutually exacerbate each other (67). Synthesizing these lines of evidence, cultivating a supportive school interactive climate and strengthening emotional bonds among teachers, peers, and students may furnish adolescents with enriched cognitive resources and emotion regulation strategies, reduce anxiety susceptibility, and consequently ameliorate sleep quality.

### 4.4 Physical exercise, anxiety

The current study revealed that PE and anxiety function as sequential mediators in the relationship between SC and SD among adolescents. PE has been widely proven to be associated with improved mental and psychological wellbeing. Conservation of Resources Theory (69) furnishes an integrative framework for elucidating this sequential mechanism, positing that SC functions as a critical social resource capable of promoting PE engagement, thereby augmenting adolescents‘ physiological and psychological resource reserves, subsequently reducing anxiety levels, and ultimately enhancing sleep health. PE facilitates a higher level of interaction between adolescents and their peer groups, thereby enhancing the social support they receive. In this process, adolescents experience a significant increase in opportunities for social engagement, while fear of social situations and concerns about negative evaluation are correspondingly reduced, effectively alleviating socially induced anxiety (70). Furthermore, due to the sensitivity characteristic of adolescence, young people often exhibit low self-esteem, feelings of inferiority, and anxiety. PE provides a means to improve body image, modify self-perceptions, and reshape interpersonal confidence during physical activity (71). Lastly, PE promotes the release of neurochemicals such as endorphins and serotonin, which exert calming and soothing effects, helps regulate autonomic nervous system balance, and reduces sympathetic nervous system excitability, thereby further alleviating anxiety (72). Empirical neuroimaging evidence has demonstrated that regular PE strengthens prefrontal cortical inhibitory control over the amygdala, dampens excessive reactivity to threat-related stimuli, and optimizes the neural substrates underlying emotion regulation (73). Synthesizing these findings, enhanced SC may satisfy adolescents' needs for peer relatedness and belongingness, thereby activating intrinsic motivation for PE engagement; regular PE subsequently alleviates anxiety stemming from academic pressure and social challenges, and the resultant reduction in anxiety levels ultimately diminishes the risk of SD. This sequential pathway elucidates the intrinsic mechanisms through which SC protects adolescent sleep health, thereby offering both a theoretical basis and actionable guidance for integrating school-based mental health services with physical education programs.

### 4.5 Heterogeneous effects of school connectedness subdimensions on adolescent sleep disorder

To further elucidate the differential mechanisms underlying the impact of SC on adolescent SD, this study disaggregated SC into its three core subdimensions: TS, CS, and SB. Although all three dimensions demonstrated significant negative total effects on SD (with total effect sizes of−0.128,−0.144, and−0.323, respectively), their mediating pathways via PE and anxiety exhibited notable distinctions. These results highlight the heterogeneous contributions of individual SC components in shaping adolescent SD.

#### 4.5.1 Teacher support: predominantly direct effects with limited mediating pathways

The total effect of TS on SD was significant (β = −0.128, *p* < 0.01), as was its direct effect (β = −0.108, *p* < 0.01). However, the associations between TS and PE (β = 0.057, *p* > 0.05) as well as between TS and anxiety (β = −0.020, *p* > 0.05) were not statistically significant. Although a serial mediating effect was detected (β = −0.126, *p* < 0.01), the proportion of the total indirect effect relative to the total effect was only 15.63%. These findings indicate that the beneficial impact of TS on SD operates primarily through a direct protective pathway, rather than indirectly by promoting PE or alleviating anxiety. A plausible underlying mechanism is that TS directly reduces the risk of SD among adolescents by providing rule-guided behavioral structuring, psychological safety, and daily routine stability.

#### 4.5.2 Classmate support: significant direct effects with minimal mediating pathways

The total effect of CS on SD (β = −0.144, *p* < 0.01) and its direct effect (β = −0.130, *p* < 0.001) were both significant. However, CS showed no statistically significant associations with PE (β = 0.024, *p* > 0.05) or anxiety (β = −0.018, *p* > 0.05). Although the serial mediating effect reached statistical significance, the total indirect effect accounted for only 9.72% of the total effect—a proportion considerably lower than that observed for SB. These results suggest that CS operates predominantly as a direct protective factor through peer companionship and social buffering, rather than via behavioral (PE) or emotional (anxiety) pathways. The underlying mechanism of CS may be more closely related to the direct relief afforded by emotional affiliation than to sequential behavioral chain transmission.

#### 4.5.3 School belonging: core protective factor with emotion-driven pathways

In contrast, SB exhibited the largest total effect (β = −0.323, *p* < 0.001) and direct effect (β = −0.178, *p* < 0.001) on SD among the three subdimensions of SC. It was significantly associated with both PE (β = 0.160, *p* < 0.001) and anxiety (β = −0.244, *p* < 0.001). The mediating effect via anxiety accounted for 39.32% of the total effect, while the total indirect effect proportion reached 44.89%. Although the serial mediation pathway (PE → anxiety) did not reach statistical significance (*p* > 0.05), the pathway “SB → anxiety → SD” was robust. These findings suggest that SB is the most protective dimension of SC, with its primary mechanism operating through the reduction of anxiety, which in turn ameliorates SD among adolescents.

In summary, the effects of the subdimensions of SC on adolescent SD are not homogeneous. SB represents the most central emotional protective pathway, whereas TS and CS primarily exert direct buffering effects without substantial indirect transmission via PE or anxiety. These findings suggest that, in intervention practices, priority should be given to enhancing students' sense of SB to effectively alleviate anxiety and consequently improve SD. In contrast, merely strengthening teacher-student or peer relationships—without the concomitant engagement of emotional regulatory mechanisms—may yield only limited protective benefits against SD.

This study reveals that SC not only exerts a direct positive effect on adolescent SD but also operates indirectly through its three core dimensions—TS, CS, and SB—to further reduce SD by promoting PE and mitigating anxiety. In the context of preventing adolescent SD, schools, as critical settings, should actively encourage and support adolescents' engagement in PE. This health-promoting behavior not only directly alleviates SD but also effectively ameliorates sleep problems arising from psychological factors such as anxiety and tension. Through regular PE, adolescents can release anxiety, enhance self-regulatory capacity, and boost self-confidence—key psychological resources that help prevent the exacerbation of SD. Accordingly, educators should foster an environment that encourages students to participate in consistent PE as part of daily school life, thereby promoting both physical and mental wellbeing and effectively managing SD. By further elucidating the relationship between SC and adolescent SD, this study uncovers the underlying mechanisms linking these constructs, thereby providing valuable insights for future research on the prevention and intervention of SD among adolescents.

### 4.6 Advantages and limitations

The present investigation examined the association between SC and SD, with particular emphasis on the mediating roles of PE and anxiety. The psychological and behavioral mechanisms underlying these interrelationships were further elucidated. By simultaneously incorporating protective and risk factors, this study enhanced the robustness of the findings and underscored their practical significance for clinical psychology practice. The results furnish actionable strategies for addressing the impact of insufficient PE and concomitant anxiety on SD among adolescents. Although this investigation effectively illuminated the underlying mechanisms linking SC to SD, several limitations warrant acknowledgment. First, the reliance on self-report measures for assessing all study variables may have compromised objectivity and accuracy; future research would benefit from integrating subjective and objective assessment methodologies to enhance reliability. Second, the cross-sectional nature of this study prevents causal conclusions. Future research should adopt longitudinal or time-series designs to identify temporal antecedents influencing adolescent SD from an SC perspective and to establish causal pathways that inform more effective prevention strategies. Third, the inclusion of only two mediating variables may have limited the model's capacity to fully explain the total effect of SC on SD. Given that adolescents spend a substantial amount of time in school, academic procrastination—as a behavioral variable closely associated with the school environment—may exert an independent influence on their sleep health. Therefore, it is recommended that future research incorporate this relevant variable into analytical frameworks. Fourth, the geographic and cultural uniqueness of the sample restricts the generalizability of the conclusions. Accordingly, future studies should be conducted across varied cultural and regional settings to enhance the external validity of the findings.

## 5 Conclusion

The present investigation aimed to examine whether the degree of SC could reduce adolescent SD through enhanced PE engagement and attenuated anxiety. The findings indicated that elevated SC exerted beneficial effects on SD among adolescents. Simultaneously, a chain mediating effect of PE and anxiety was observed in the relationship between SC and SD. By analyzing the interrelationships among SC, PE, anxiety, and SD, this investigation extended existing research on the association between SC and SD, offering novel insights into the multifaceted pathways through which SC influences SD. Incorporating the social ecological factor of SC into the analytical framework of adolescent SD has expanded the perspective on the social determinants of sleep health. The use of a chain mediation model enabled a deeper investigation into the mechanisms connecting adolescent SC to SD, thereby enriching the theoretical understanding of PE's psychological effects and generating novel hypotheses for future research. In practical terms, this study provides actionable intervention targets for school-based mental health education. Furthermore, it emphasizes preventive intervention, suggesting that early intervention can be implemented through modifiable variables—such as sense of belonging, amount of PE, and anxiety levels—before sleep problems become severe. Furthermore, this study recommends that school policy makers consider adolescents' social interactions, behavioral patterns, and sense of SB when designing individualized PE programs, and take into account the bidirectional interplay between PE and psychological wellbeing as well as its potential moderating factors.

## Data Availability

The raw data supporting the conclusions of this article will be made available by the authors, without undue reservation.

## References

[1] Hansen SL Capener D Daly C. Adolescent sleepiness: causes and consequences. Pediatr Ann. (2017) 46:e340–4. doi: 10.3928/19382359-20170816-01 28892550

[2] Zammit GK Weiner J Damato N Sillup GP McMillan CA. Quality of life in people with insomnia. Sleep. (1999) 22(Suppl 2):S379–85. 10394611

[3] Prono F Bernardi K Ferri R Bruni O. The role of vitamin D in sleep disorders of children and adolescents: a systematic review. Int J Mol Sci. (2022) 23:1430. doi: 10.3390/ijms23031430 35163353 PMC8835880

[4] Yang H Luan L Xu J Xu X Tang X Zhang X. Prevalence and correlates of sleep disturbance among adolescents in the eastern seaboard of China. BMC Public Health. (2024) 24:1003. doi: 10.1186/s12889-024-18564-0 38600538 PMC11008010

[5] Liang M Guo L Huo J Zhou G. Prevalence of sleep disturbances in Chinese adolescents: a systematic review and meta-analysis. PLoS ONE. (2021) 16:e247333. doi: 10.1371/journal.pone.0247333 33661914 PMC7932116

[6] Chai R Bian W. Adolescent sleep and its disruption in depression and anxiety. Front Neurosci-Switz. (2024) 18:1479420. doi: 10.3389/fnins.2024.1479420 39575099 PMC11578994

[7] Crowley SJ Wolfson AR Tarokh L Carskadon MA. An update on adolescent sleep: new evidence informing the perfect storm model. J Adolescence. (2018) 67:55–65. doi: 10.1016/j.adolescence.2018.06.001 29908393 PMC6054480

[8] Tarokh L Guiterrez Herrera C. Adolescent sleep disruption: implications for psychiatric morbidity. Biol Psychiat. (2025) 98:854–62. doi: 10.1016/j.biopsych.2025.08.010 40902697

[9] Meneo D Baldi E Benz F Fabris MA. A biopsychosocial approach to sleep health during puberty: Individual and contextual aspects and the role of gender differences. a narrative review. Cogent Mental Health. (2025) 4:2541697. doi: 10.1080/28324765.2025.2541697 41262959 PMC12442616

[10] Johri K Pillai R Kulkarni A Balkrishnan R. Effects of sleep deprivation on the mental health of adolescents: a systematic review. Sleep Science and Practice. (2025) 9:9. doi: 10.1186/s41606-025-00127-w

[11] Colrain IM Baker FC. Changes in sleep as a function of adolescent development. Neuropsychol Rev. (2011) 21:5–21. doi: 10.1007/s11065-010-9155-5 21225346 PMC7543715

[12] Force APAZ. Are zero tolerance policies effective in the schools? An evidentiary review and recommendations. Am Psychol. (2008) 63:852–62. doi: 10.1037/0003-066X.63.9.852 19086747

[13] Ni P Zheng Z Wu R. The concept of school connection and related research brief introduction. Friends of humanity. (2019):2.

[14] Bat-Pitault F Viorrain M Da Fonseca D Charvin I Guignard-Perret A Putois B . Adolescent sleep disorders associated with school absenteeism: the child and adolescent psychiatrist is often crucial for effective management in sleep consultation. Encephale. (2019) 45:82–9. doi: 10.1016/j.encep.2018.06.006 30122297

[15] Phillips SR Ward T Carmiol-Rodriguez P Coker T Carlin K. 0357 school connectedness and sleep health during adolescence. Sleep. (2025) 48(Supplement_1):A155-A156. doi: 10.1093/sleep/zsaf090.0357

[16] Shea A. Cognitive-Behavioral Model. In: Encyclopedia of Feeding and Eating Disorders. Edited by Wade T. Singapore: Springer Singapore (2017):145-150. doi: 10.1007/978-981-287-104-6_205

[17] Bacaro V Carpentier L Crocetti E. Sleep well, study well: a systematic review of longitudinal studies on the interplay between sleep and school experience in adolescence. Int J Environ Res Public Health. (2023) 20:4829. doi: 10.3390/ijerph20064829 36981738 PMC10049641

[18] Diggs D Deniz E Toseeb U. School connectedness as a protective factor between childhood adversity and adolescent mental health outcomes. Dev Psychopathol. (2025) 37:1355–73. doi: 10.1017/S0954579424001184 39506487

[19] Brodar KE La Greca AM Hysing M Llabre MM. Stressors, repetitive negative thinking, and insomnia symptoms in adolescents beginning high school. J Pediatr Psychol. (2020) 46:40–52. doi: 10.1093/jpepsy/jsaa064 32968794

[20] Du Y Wan X Xu J Feng H. The association between social anxiety and sleep among young people: a systematic review and meta-analysis. Child Psychiatry Hum Dev. (2026). doi: 10.1007/s10578-026-02003-9 41995981

[21] Caspersen CJ Powell KE Christenson GM. Physical activity, exercise, and physical fitness: definitions and distinctions for health-related research. Public Health Rep. (1985) 100:126–31. 3920711 PMC1424733

[22] McLoughlin GM Graber KC. The contribution of physical education to physical activity within a comprehensive school health promotion program. Res Q Exercise Sport. (2021) 92:669–79. doi: 10.1080/02701367.2020.1765952 32809917

[23] Zhou X Zhang M Chen L Li B Xu J. The effect of peer relationships on college students' behavioral intentions to be physically active: The chain-mediated role of social support and exercise self-efficacy. PLoS ONE. (2025) 20:e320845. doi: 10.1371/journal.pone.0320845 40344563 PMC12064194

[24] Tiwari BB Matta J Thomsen MR Da L Thapa K Shen Y . Association between adverse childhood experiences and mental health outcomes among adolescents and young adults. Front Public Health. (2025) 13:1582693. doi: 10.3389/fpubh.2025.1582693 40756404 PMC12313673

[25] Martinsone B Gērmane E Neves-McCain JR. Adolescents' perceived school climate, self-reported mental health, and frequency of physical activity: associations and gender differences. Front Child Adolesc Psychiatry. (2025) 4:1674304. doi: 10.3389/frcha.2025.1674304 41625263 PMC12855475

[26] Ryan RM Deci EL. Self-determination theory: Basic psychological needs in motivation, development, and wellness. In: Self-determination theory: Basic psychological needs in motivation, development, and wellness. New York, NY, US: The Guilford Press; 2017: 756, 756. doi: 10.1521/978.14625/28806

[27] Tang S Chen H Lu T Tao B Yan J. The effects of physical exercise on adolescents' school adjustment and path analysis—evidence from the China education panel survey. Behav Sci. (2025) 15:1602. doi: 10.3390/bs15121602 41463948 PMC12729406

[28] Wunsch K Kasten N Fuchs R. The effect of physical activity on sleep quality, well-being, and affect in academic stress periods. Nat Sci Sleep. (2017) 9:117–26. doi: 10.2147/NSS.S132078 28490911 PMC5414656

[29] Kredlow MA Capozzoli MC Hearon BA Calkins AW Otto MW. The effects of physical activity on sleep: a meta-analytic review. J Behav Med. (2015) 38:427–49. doi: 10.1007/s10865-015-9617-6 25596964

[30] Hou L-J Geng Y-X Li K Huang Z-Y Ma L-Q. Research on the role of dopamine in regulating sleep and wakefulness through exercise. Prog Biochem Biophys. (2025) 52:88–98. doi: 10.16476/j.pibb.2024.0157

[31] Griffiths PE Tabery J. Chapter Three—Developmental Systems Theory: what does it explain, and how does it explain it? In: Lerner RM Benson JB, editors. Adv Child Dev Behav. (2013) 44:65–9. doi: 10.1016/B978-0-12-397947-6.00003-9 23834002

[32] Lahousen T Kapfhammer HP. Anxiety disorders - clinical and neurobiological aspects. Psychiat Danub. (2018) 30:479–90. doi: 10.24869/psyd.2018.479 30439809

[33] de Haan S. Bio-psycho-social interaction: an enactive perspective. Int Rev Psychiatr. (2021) 33:471–7. doi: 10.1080/09540261.2020.1830753 33236671

[34] Zhang P Yuan Y Wang M Ding L Zhang J Qiu S. The relationship between social exclusion and social anxiety among Chinese college students: a chain mediation role of fear of negative evaluation and rejection sensitivity. Curr Psychol. (2025) 44:7181–92. doi: 10.1007/s12144-025-07689-z

[35] Lou W. Research on the Influence of Peer Pressure on Adolescents. Lect Notes Educ Psychol Public Media. (2023) 8:163–9. doi: 10.54254/2753-7048/8/20230090

[36] Zhang Z Li X Juan G Qi C. The influence of teacher care on middle school students' social–emotional competence: evidence from the China Education Panel Survey. (2013–2014). Front Psychol. (2026) 17:1748385. doi: 10.3389/fpsyg.2026.1748385 41835893 PMC12982437

[37] Pikulski PJ Pella JE Casline EP Hale AE Drake K Ginsburg GS. School connectedness and child anxiety. J Psychol Couns Sch. (2020) 30:13–24. doi: 10.1017/jgc.2020.3

[38] Feiss R Dolinger SB Merritt M Reiche E Martin K Yanes JA . A systematic review and meta-analysis of school-based stress, anxiety, and depression prevention programs for adolescents. J Youth Adolescence. (2019) 48:1668–85. doi: 10.1007/s10964-019-01085-0 31346924 PMC7548227

[39] Crowe K Spiro-Levitt C. Sleep-related problems and pediatric anxiety disorders. Psychiat Clin N Am. (2024) 47:213–28. doi: 10.1016/j.psc.2023.06.014 38302208

[40] Ross JA Van Bockstaele EJ. The locus coeruleus-norepinephrine system in stress and arousal: unraveling historical, current, and future perspectives. Front Psychiatry. (2021) 11:601519. doi: 10.3389/fpsyt.2020.601519 33584368 PMC7873441

[41] Chellappa SL Aeschbach D. Sleep and anxiety: From mechanisms to interventions. Sleep Med Rev. (2022) 61:101583. doi: 10.1016/j.smrv.2021.101583 34979437

[42] Walker WH Walton JC DeVries AC Nelson RJ. Circadian rhythm disruption and mental health. Transl Psychiat. (2020) 10:28. doi: 10.1038/s41398-020-0694-0 32066704 PMC7026420

[43] Iwon K Skibinska J Jasielska D Kalwarczyk S. Elevating subjective well-being through physical exercises: an intervention study. Front Psychol. (2021) 12:702678. doi: 10.3389/fpsyg.2021.702678 34975608 PMC8719442

[44] Gontkovsky ST. Biological psychology: an introduction to behavioral, cognitive, and clinical neuroscience, third edition. J Neuropsychiatry Clin Neurosci. (2004) 16:122–3. doi: 10.1176/jnp.16.1.122

[45] Belcher BR Zink J Azad A Campbell CE Chakravartti SP Herting MM. The Roles of Physical Activity, Exercise, and Fitness in Promoting Resilience During Adolescence: Effects on Mental Well-Being and Brain Development. Biol Psychiatry Cogn Neurosci Neuroimaging. (2021) 6:225–37. doi: 10.1016/j.bpsc.2020.08.005 33067166 PMC7878276

[46] Tian J Yu H Austin L. The effect of physical activity on anxiety: the mediating role of subjective well-being and the moderating role of gender. Psychol Res Behav Manag. (2022) 15:3167–78. doi: 10.2147/PRBM.S384707 36324422 PMC9621221

[47] Yan C Liu Y Xiao T Zhang T. Psychological resilience and emotional distress as chain mediators between physical activity and short video dependency among adolescents. J Genet Psychol. (2026) 187:1-16. doi: 10.1080/00221325.2026.2625059 41661023

[48] Yan C Liu Y Xiao T Pan M Zhang T He Z . Parental marital conflict and adolescent anxiety: the moderating roles of physical activity and gender. Psychol Psychother-T. (2026) 99:484–500. doi: 10.1111/papt.70037 41548139

[49] Cotman CW Berchtold NC Christie LA. Exercise builds brain health: key roles of growth factor cascades and inflammation. Trends Neurosci. (2007) 30:464–72. doi: 10.1016/j.tins.2007.06.011 17765329

[50] Lam PH. An extension to the stress-buffering model: timing of support across the lifecourse. Brain Behav Immun Health. (2024) 42:100876. doi: 10.1016/j.bbih.2024.100876 39430880 PMC11490906

[51] Yu CP Zhang W Zheng Y. Relationship between adolescents' gratitude and problem behavior: the mediating role of school connectedness. Psychol Dev Educ. (2011) 27:425–33.

[52] Hair JF Black WC Babin BJ Anderson RE. Multivariate data analysis. 7th ed. Upper Saddle River (NJ): Prentice Hall (2009).

[53] Liang DQ. The stress level of college students and its relationship with physical exercise. Chin Ment Health J. (1994) 8:5–6.

[54] Gong X Xie X-Y Xu R Luo Y-J. Psychometric Properties of the Chinese Versions of DASS-21 in Chinese College Students. Chin J Clin Psychol. (2010) 18:443–6. doi: 10.16128/j.cnki.1005-3611.2010.04.020

[55] Buysse DJ Reynolds CR Monk TH Berman SR Kupfer DJ. The Pittsburgh sleep quality index: a new instrument for psychiatric practice and research. Psychiat Res. (1989) 28:193–213. doi: 10.1016/0165-1781(89)90047-4 2748771

[56] Podsakoff PM MacKenzie SB Lee JY Podsakoff NP. Common method biases in behavioral research: a critical review of the literature and recommended remedies. J Appl Psychol. (2003) 88:879–903. doi: 10.1037/0021-9010.88.5.879 14516251

[57] Hayes AF. Partial, conditional, and moderated moderated mediation: Quantification, inference, and interpretation. Commun Monogr. (2018) 85:4–40. doi: 10.1080/03637751.2017.1352100

[58] Berkovits I Hancock GR Nevitt J. Bootstrap resampling approaches for repeated measure designs: relative robustness to sphericity and normality violations. Educ Psychol Meas. (2000) 60:877–92. doi: 10.1177/00131640021970961

[59] Bao Z Chen C Zhang W Jiang Y Zhu J Lai X. School connectedness and Chinese adolescents' sleep problems: a cross-lagged panel analysis. J School Health. (2018) 88:315–21. doi: 10.1111/josh.12608 29498062

[60] Bronfenbrenner U. Ecological systems theory. In:Kazdin AE, editor. Encyclopedia of Psychology. Vol. 3. Washington (DC): American Psychological Association (2000). p. 129–133. doi: 10.1037/10518-046

[61] Boccalaro IL Rillosi E Ramirez-Plascencia O De Luca R Mahoney CE. A narrative review on oxytocin at the intersection of sleep, stress, and social behavior. Front Neurosci. (2026) 20:1745281. doi: 10.3389/fnins.2026.1745281 42093825 PMC13139196

[62] Dowd AJ Schmader T Sylvester BD Jung ME Zumbo BD Martin LJ . Effects of social belonging and task framing on exercise cognitions and behavior. J Sport Exercise Psy. (2014) 36:80–92. doi: 10.1123/jsep.2013-0114 24501146

[63] Cohen S Wills TA. Stress, social support, and the buffering hypothesis. Psychol Bull. (1985) 98:310–35. doi: 10.1037/0033-2909.98.2.310 3901065

[64] Jung A Park J Kim H. Physical Activity for Prevention and Management of Sleep Disturbances. Sleep Med Res. (2020) 11:15–8. doi: 10.17241/smr.2020.00542

[65] Raniti M Rakesh D Patton GC Sawyer SM. The role of school connectedness in the prevention of youth depression and anxiety: a systematic review with youth consultation. BMC Public Health. (2022) 22:2152. doi: 10.1186/s12889-022-14364-6 36424575 PMC9694921

[66] Dixon ML Thiruchselvam R Todd R Christoff K. Emotion and the prefrontal cortex: an integrative review. Psychol Bull. (2017) 143:1033–81. doi: 10.1037/bul0000096 28616997

[67] Nguyen VV Zainal NH Newman MG. Why Sleep is Key: Poor Sleep Quality is a Mechanism for the Bidirectional Relationship between Major Depressive Disorder and Generalized Anxiety Disorder Across 18 Years. J Anxiety Disord. (2022) 90:102601. doi: 10.1016/j.janxdis.2022.102601 35850001 PMC9945467

[68] Liu D Lei G Qiu L Deng H Liu S Dang Y . Rumination and insomnia in Chinese adolescents with mood disorders: the mediating role of anxiety. (2023).

[69] Holmgreen L Tirone V Gerhart J Hobfoll S. Conservation of resources theory. In:Zeigler-Hill V Shackelford TK, editors. Encyclopedia of Personality and Individual Differences. Cham: Springer (2017). p. 443–457. doi: 10.1002/9781118993811.ch27

[70] Seabrook EM Kern ML Rickard NS. Social networking sites, depression, and anxiety: a systematic review. JMIR Ment Health. (2016) 3:e50. doi: 10.2196/mental.5842 27881357 PMC5143470

[71] Iancu I Bodner E Ben-Zion IZ. Self esteem, dependency, self-efficacy and self-criticism in social anxiety disorder. Compr Psychiat. (2015) 58:165–71. doi: 10.1016/j.comppsych.2014.11.018 25556952

[72] Di Liegro CM Schiera G Proia P Di Liegro I. Physical activity and brain health. Genes. (2019) 10:720. doi: 10.3390/genes10090720 31533339 PMC6770965

[73] Chen Y Chen C Martínez RM Etnier JL Cheng Y. Habitual physical activity mediates the acute exercise-induced modulation of anxiety-related amygdala functional connectivity. Sci Rep. (2019) 9:19787. doi: 10.1038/s41598-019-56226-z 31875047 PMC6930267

