# Correction: The chain mediating role of physical exercise and anxiety in the relationship between school connectedness and sleep disorders among adolescents

**DOI:** 10.3389/fpubh.2026.1947390

**Published:** 2026-08-11

**Authors:** Xiaozhen Wu, Ning Wang, Juyan Fang, Yang Liu, Binbin Zhao, Chunhui Zhou

**Affiliations:** 1 Fuyang Normal University, Fuyang, China; 2 Chengdu Vocational University of the Arts, Chengdu, China; 3 Jishou University, Jishou, China; 4 Chengdu Experimental Middle School, Chengdu, China; 5 Shenyang Normal University, Shenyang, China; 6 Zhangjiajie Institute of Aeronautical Engineering, Zhangjiajie, China

**Keywords:** adolescents, anxiety, physical exercise, school connectedness, sleep disorders

In the published version, affiliation 2 for author Ning Wang is incorrectly listed as “Guangxi MINZU University, Nanning, China”. The correct affiliation 2 should be “Chengdu Vocational University of the Arts, Chengdu, China”.

The original version of this article has been updated.

